# Robust Displacement Sensing by Direct‐Current Triboelectric Nanogenerator Via Intelligent Waveform Recognition

**DOI:** 10.1002/advs.202204694

**Published:** 2022-12-04

**Authors:** Keren Dai, Xuyi Miao, Wenling Zhang, Xiaohua Huang, He Zhang, Sang‐Woo Kim

**Affiliations:** ^1^ School of Mechanical Engineering Nanjing University of Science and Technology Nanjing 210094 China; ^2^ School of Advanced Materials Science and Engineering Sungkyunkwan University (SKKU) Suwon 16419 Republic of Korea

**Keywords:** direct‐current triboelectric nanogenerators, displacement sensing, environmental robustness, intelligent signal processing

## Abstract

A triboelectric nanogenerator (TENG) facilitates the advancement of self‐powered displacement sensors, which are important for many autonomous intelligent microsystems. However, the amplitude‐based displacement sensing of conventional TENG‐based sensors still suffers significantly from varying charge densities in harsh environments. Benefiting from the combination of intelligent signal processing algorithms and direct‐current TENG sensors, this study proposes an environmentally robust character‐based displacement sensing method that eliminates the influences of varying charge density in principle. The experimental results show that under drastically changing air humidity and other harsh environments, the sensing of threshold and maximum displacement has far superior consistency and stability than that of traditional amplitude‐based TENG sensors, providing a novel route to realize reliable self‐powered displacement sensing in environment‐variable applications.

## Graphical abstract

An environmentally robust displacement sensing method for direct‐current triboelectric nanogenerator sensors is proposed. The displacement is calibrated based on the character of inflection point on the output waveform. So, the influences of varying charge density due to the drastically changing air humidity and other harsh environments can be eliminated in principle.

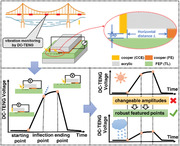

## 1 Introduction

In recent years, near‐zero‐power microsystems or self‐powered microsystems continue to bring revolutionary advancements to the military,^[^ ^1^ ^]^ the internet of things,^[^ ^2^, ^3^ ^]^ medicine,^[^ ^4^, ^5^ ^]^ and many other fields. In particular, self‐powered sensors are key devices for these smart microsystems.^[^ ^6^ ^]^ The triboelectric nanogenerator (TENG) technology enabled the recent round of increasing development of self‐powered sensors from the time it was invented. A large variety of TENG‐based self‐powered sensors has been proposed,^[^ ^7^, ^8^ ^]^ significantly promoting the industrial advancement of autonomous intelligent microsystems. Most TENG‐based sensors can be divided into two categories in principle: frequency‐based and amplitude‐based sensors, both of which have been widely used. The frequency‐based sensors have been used as pulse,^[^ ^9^, ^10^ ^]^ heart rate,^[^ ^11^ ^]^ rotating speed sensors,^[^ ^12^, ^13^ ^]^ and so on, while the amplitude‐based sensors have been used as displacement,^[^ ^14^, ^15^ ^]^ acceleration,^[^ ^16^, ^17^ ^]^ stress,^[^ ^18^, ^19^ ^]^ and force sensors,^[^ ^20^, ^21^, ^22^, ^23^ ^]^ and so on. Among them, the displacement sensors not only have great potential in specific industrial scenarios such as bridge vibration monitoring^[^ ^24^ ^]^ but also form the technical basis for acceleration and force sensors, which are essentially realized by sensing the acceleration‐relevant or force‐relevant displacements.

However, there is a concern that the robustness of the amplitude‐based TENG sensor is not as good as that of the frequency‐based ones because the voltage or current amplitude of the TENG‐based sensor is proportional to the surface charge density of its friction material. The humidity,^[^ ^25^, ^26^ ^]^ temperature,^[^ ^27^, ^28^ ^]^ abrasion,^[^ ^29^ ^]^ and other factors all have non‐negligible influences on the surface charge density, and these non‐ideal factors cannot be completely avoided in practical applications. For example, in a rainy or hot day, the surface charge density is lower than normal due to increased humidity or temperature. In addition, as friction wear increases, the surface charge density also decreases. Therefore, the sensing robustness arising from surface charge density fluctuations is a major difficulty restricting further practical development of displacement sensors and other amplitude‐based TENG sensors. Fortunately, the development of direct‐current TENG (DC‐TENG)^[^ ^30^, ^31^, ^32^ ^]^ provides a new way to solve the problem of sensing robustness. Different from the output waveform of traditional TENG, the output waveform of DC‐TENG, especially the air‐breakdown DC‐TENG,^[^ ^33^ ^]^ has distinct characteristics, which are two successive phases of rising and constant. Previous theoretical studies^[^ ^34^ ^]^ have shown that the inflection point between the rising and constant phases of the output waveform is a result of the air‐breakdown effect and makes a contribution to the superior high output power of DC‐TENG. However, previous studies have only discussed the significance of this output characteristic of DC‐TENG as the high‐performance power source but didn't consider the advantage of the inherent robustness of self‐powered sensors. Actually, as the displacement of the inflection point is only determined by fixed structural parameters of the DC‐TENG device and independent of environmental factors, they can be used as the reference. Therefore, robust displacement sensing can be realized via the output waveform characteristics of the DC‐TENG sensor instead of the amplitude as it is discussed in this paper. This work is expected to provide new inspiration for the design of TENG‐based displacement sensors and promote the application of such devices in more complex and changeable environments.

In this paper, the robust displacement sensing by DC‐TENG via intelligent waveform recognition is introduced in detail. In the following sections of this paper, first, the robust displacement sensing mechanism is proposed and a theoretical explanation is provided as to why its sensing results are independent of variable environmental parameters; then, the signal processing algorithm for its waveform recognition is proposed, which makes the DC‐TENG device a truly operational robust displacement sensing system; finally, the experimental robust sensing performances are presented and discussed, verifying its stable sensing resulting under wide‐range variable air humidity and surface roughness.

## 2 Results and Discussion

### 2.1 Robust Sensing Mechanism

**Figure 1:**
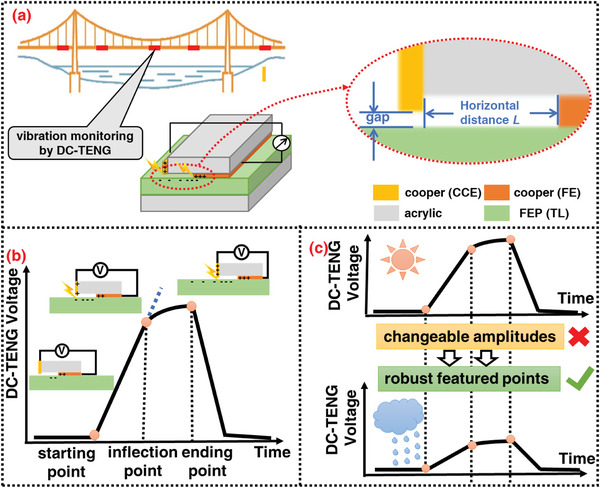
The robust displacement sensing mechanism of the DC‐TENG sensor. a) Application scenario of the DC‐TENG sensor and its structure; b) relationship between waveform characteristics and displacement; c) invariant waveform characteristics under different environments.

**Figure** **1**a shows the proposed displacement sensor based on air‐breakdown DC‐TENG, and it is aimed at bridge vibration monitoring and other similar scenarios, where the maximum value of the displacement is very important, especially whether it exceeds the specified threshold. In these scenarios, environmental factors such as humidity and temperature are not controllable or predictable, which may cause fluctuation of surface charge density for TENG sensors. The structure of the proposed sensor in Figure 1a is the standard structure of the air breakdown DC‐TENG that is used in previous literature,^[^ ^33^ ^]^ in which the charge is generated by friction between the frictional electrode (FE) and triboelectric layer (TL) and consumed by air breakdown between the charge collecting electrode (CCE) and TL.

The robust sensing mechanism of this DC‐TENG displacement sensor can be attributed to the inherent relationship between the feature points of its output waveform and its structural parameters. As shown in Figure 1b, for a classic air breakdown DC‐TENG, its most important structural parameter is the horizontal distance *L* between the FE and CCE. The output waveform of the DC‐TENG sensor can be divided into two different stages according to the horizontal distance *L*: when the displacement is less than *L*, the air breakdown has not yet occurred, and the output increases almost linearly with the displacement; when the displacement exceeds *L*, the air breakdown occurs continuously, and the output gradually tends to be flat. Therefore, the displacement at the inflection point dividing these two stages is approximately the same as the horizontal distance *L* and is nearly unaffected by other environmental factors. Through intelligent signal processing, it can be determined whether the displacement exceeds the threshold when the inflection point of the waveform (also called the threshold point) is found or not. Moreover, if the motion model is known (e.g., uniform motion, uniformly accelerated motion, etc.), the displacement corresponding to the inflection point can be used as a reference, and the sensing of maximum displacement corresponding to the ending point of the output waveform can be realized.

This displacement sensing method is robust in principle. As shown in Figure 1c, when the environment changes from sunny to rainy, or the air humidity increases, the surface charge density of the TENG decreases. Therefore, the amplitude of the output waveform also decreases as a whole. If displacement sensing is traditionally determined by amplitude, a large and unpredictable bias occurs. Fortunately, as long as the surface charge density is not too low, air breakdown can still occur, and the waveform characteristics of the sensor output are almost unchanged as the displacement of the inflection point only depends on the special structure parameter of DC‐TENG. Thus, the maximum displacement can be estimated based on the starting, inflection, and ending points, avoiding the influence of the variable environmental factors. Therefore, the key to realizing such robust sensing is the reliable identification of these feature points as the reliable reference via intelligent algorithms, which is presented as follows.

### 2.2 Signal Processing Algorithm

**Figure 2:**
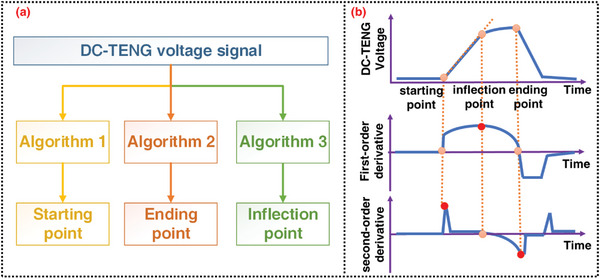
Signal processing and feature point recognition algorithms of the proposed DC‐TENG displacement sensor. a) Algorithm architecture and b) waveform diagram illustrating the algorithm mechanism.

According to the aforementioned robust sensing mechanism, accurate identification of the starting, inflection, and ending points of the output waveform is the key to achieving accurate displacement sensing. Thus, a set of signal processing and feature point recognition algorithms is proposed for the DC‐TENG displacement sensor. For the simplicity of the algorithm, the movement is assumed to be uniform in this study, and the specific algorithm is shown in Figure S1, Supportng Information. The basic idea of the algorithm is similar to other forms of motion, though with more complex calculations. The starting point is identified with two basic principles. First, the amplitude at the starting point is very close to 0; second, the starting point is a concave point, which means that its second derivative is a local maximum point. Thus, the detailed steps for the identification of the starting point *t*_start_ are summarized in Algorithm 1 (**Figure** **2**; Figure S1, Supporting Information). In Step 1, signal filtering is carried out for denoising; in Step 2, the time interval *T*_zero_ around the starting point of the filtered signal is determined, where the signal amplitude is in the range of [−*δ*,*δ*]. Here, *δ* is a preset threshold with a very small value, which is determined by the noise strength. In Step 3, within the time interval *T*_zero_, the largest maximum point of the second‐order derivative of the filtered signal is determined as *t*_start_.

The ending point is identified based on two basic principles. First, the ending point is at the end of the constant stage of the signal. During the constant stage, the signal amplitude is always very close to its maximum amplitude. Second, the ending point is a convex point, which means that its second derivative is a local minimum point. Thus, the detailed steps for the identification of the ending point *t*_end_ are summarized in Algorithm 2 (Figure 2; Figure S1, Supporting Information). In Step 1, signal filtering is carried out for denoising; in Step 2, the time interval *T*_max_ is determined when the signal amplitude is larger than *η*% of its maximum value. Here, *η* is a preset threshold value, 0 < *η* < 1. In Step 3, within the time interval *T*_max_, the last minimum point of the second‐order derivative of the filtered signal is determined as *t*_end_.

The algorithm for identifying the inflection points is more complicated than that for the starting and ending points. It can be divided into two sub‐algorithms: the first sub‐algorithm aims to identify the possible inflection points; the second sub‐algorithm aims to determine whether the possible point is a real inflection point. The principle of identifying the possible inflection point is to identify the point with the largest first‐order derivative, because experimental data show that the signal before the inflection point is not completely linear but slightly up‐concave due to the continuous increase of friction‐generated charge density during the early sliding stage of DC‐TENG. The determination of whether a possible inflection point is a real inflection point or not is based on two criteria. The first criterion is that the first derivative of the signal should be significantly different before and after the possible inflection point; the second criterion is that there should be a significant gap between the inflection and ending points. If both these two criteria are not met, it can be considered that the possible inflection point is not a real one. In other words, the maximum displacement is less than the threshold displacement of the sensor. The detailed steps for the identification of the inflection point *t*_inflect_ are summarized in Algorithm 3 (Figure 2; Figure S1, Supporting Information). In Step 1, signal filtering is carried out for denoising. In Step 2, the maximum point *t*_inflect_ for the first‐order derivative of the filtered signal is determined as a possible inflection point. In Step 3, the time interval Δ*t* before and after the possible inflection point, respectively, is considered to calculate the derivatives at the left and right side of the possible inflection points,

(1) $$y_{d,\mathrm{left}}\;\left(t_{\mathrm{inflect}}\right)=\frac{V\left(t_{\mathrm{inflect}}\right)-V\left(t_{\mathrm{inflect}}-\Delta t\right)}{\Delta t}$$

(2) $$y_{d,\mathrm{right}}\;\left(t_{\mathrm{inflect}}\right)=\frac{V\left(t_{\mathrm{inflect}}+\Delta t\right)-V\left(t_{\mathrm{inflect}}\right)}{\Delta t}$$

where *V*(*t*) is the output voltage from DC‐TENG. If $\frac{y_{d,\mathrm{left}}\left(t_{\mathrm{inflect}}\right)}{y_{d,\mathrm{right}}\left(t_{\mathrm{inflect}}\right)}>k_{1}$, where *k*_1_ is a preset coefficient, *t*_inflect_ is the real inflection point; if not, Step 4 is executed. In Step 4, if $\frac{t_{\mathrm{end}}-t_{\mathrm{inflect}}}{t_{\mathrm{end}}-t_{\mathrm{start}}}>k_{2}$, where *k*_2_ is a preset coefficient, then *t*_inflect_ is the real inflection point; if not, no inflection point is found, and the maximum displacement is less than the threshold displacement of the sensor.

### 2.3 Robust Threshold Displacement Sensing

To verify the proposed displacement sensing method, the recognition of inflection points in the output waveform is first experimentally tested, by performing signal processing on the experimental output waveform. The threshold displacement of the tested DC‐TENG sensor is approximately equal to the horizontal distance between its CCE and FE, which is 15 mm.

**Figure 3:**
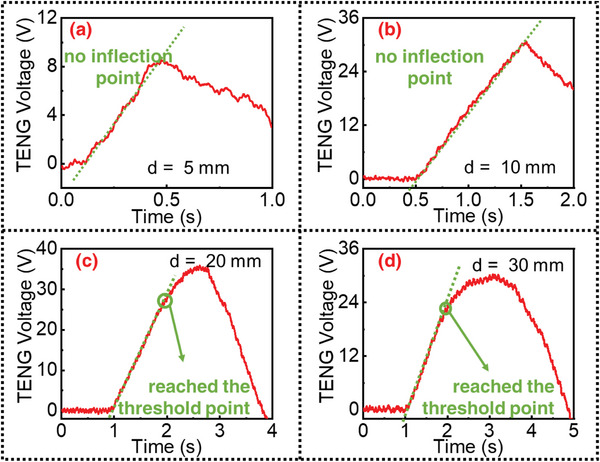
Processing result of the algorithm for the threshold displacement point. a) Maximum displacement is 5 mm, lower than the threshold; b) maximum displacement is 10 mm, lower than the threshold; c) maximum displacement is 20 mm, higher than the threshold; d) maximum displacement is 30 mm, higher than the threshold.

As shown in **Figure** **3**a,b, when the maximum displacement of the DC‐TENG sensor is 5 and 10 mm, respectively, which is less than the threshold displacement, the output signal of the sensor is almost a linear curve. For these cases, the proposed algorithm correctly identifies that there is no inflection point in the sensing waveform; and thus, determines that the maximum displacements for these cases are still lower than the sensor's threshold displacement. In contrast, as shown in Figure 3c,d, when the maximum displacement of the DC‐TENG sensor is 20 and 30 mm, respectively, which is higher than the threshold displacement, the typical inflection point of DC‐TENG waveform between a linear stage and a constant stage can be effectively recognized. In these cases, the inflection points are recognized by the algorithm at 1.72 and 1.7 s, respectively. That is, the sensor correctly estimates that the maximum displacement exceeds the threshold displacement. The sensing waveforms in Figure 3 are obtained by simple low‐pass filtering of the raw voltage signals of the DC‐TENG sensor measured by the electrometer, to suppress non‐ideal high‐frequency noises. These measured raw voltage signals are presented in Figure S2, Supporting Information.

It is more important to verify the robustness of the proposed displacement sensing method. In this study, two robustness tests were conducted to verify the environmental reliability of the proposed DC‐TENG sensors. First, to simulate different weather conditions under practical working scenarios, the DC‐TENG sensor was tested under varying air humidity. Second, to simulate different abrasion and aging situations under practical working scenarios, the DC‐TENG sensors with different plasma treatment power were tested. All the detailed information regarding the two robustness tests is presented in the Experimental Section; Figures S3–S6, Supporting Information.

**Figure 4:**
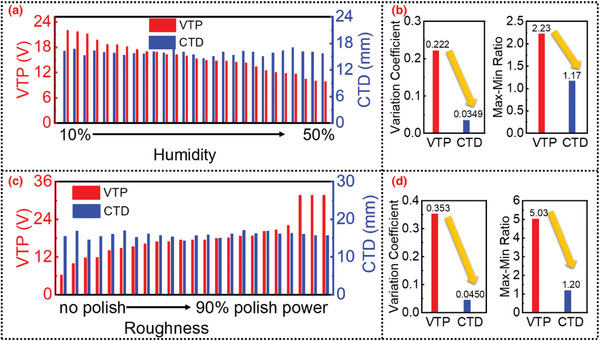
Robust sensing performances of the DC‐TENG sensor. a) The voltage at threshold point (VTP) and character‐based threshold displacement (CTD) under varying air humidity; b) statistical analysis under varying air humidity; c) VTP and the CTD under variable roughness; d) statistical analysis under variable roughness.

To make a reasonable comparison with the traditional amplitude‐based sensors, the real threshold displacement point is determined by the standard commercial sensor, and the output voltage from the TENG at the real threshold displacement point is regarded as VTP (voltage at threshold point). The raw data is shown in Figure S7, Supporting Information. Through the threshold point identification algorithm, the character‐based threshold displacements (CTD) are obtained. By comparing the stability of the VTP and CTD, the robustness of the two different displacement sensing methods can be evaluated. As shown in **Figure** **4**a, when the air humidity changes from 10% to 50%, the VTP changes significantly, ranging from ≈20 to 10 V. In contrast, the CTD is relatively stable, always ≈15 mm, which is also the designed threshold displacement for a DC‐TENG sensor. To further confirm the stability of the proposed algorithm, the air humidity is increased to 60% at a rainy day. The output waveform still retains its characteristics, and the displacement estimation results obtained by our algorithm are consistent (as shown in Figure S8, Supporting Information). Figure 4b provides the statistical analysis for the aforementioned stability comparison. The variation coefficient can be defined as,

(3) $$C=\frac{\sigma }{\mu }$$

where $\mu =\frac{1}{N}\;\sum_{n=1}^{N}D\left(n\right)$ is the average of *D*(*n*), *D*(*n*) is a series of *N* data for the robustness experiments, and $\sigma =\sqrt{\frac{\sum_{n=1}^{N}{\left(D(n)-\mu \right)}^{2}}{N}}$ is the standard deviation.

The max–min ratio can be defined as,

(4) $$M=\frac{\max\left(D\left(n\right)\right)}{\min\left(D\left(n\right)\right)}$$

Compared with the traditional amplitude‐based sensor, the proposed robust displacement sensing method can reduce the variation coefficient from 22.2% to 3.49% and reduce the max–min ratio from 2.23 to 1.17. These data credibly demonstrate the robustness of the DC‐TENG displacement sensor under drastically varying humidity. Similarly, the robustness to different roughness is significantly improved. As shown in Figure 4c, when the plasma treatment power changes in the range of 0–90%, the VTP changes more significantly, ranging from ≈5 to over 30 V, while the CTD is still always ≈15 mm. The statistics in Figure 4d show that the proposed robust displacement sensing method reduces the variation coefficient from 35.3% to 4.5% and reduces the max–min ratio from 5.03 to 1.20. The plasma treatment provides a way to evaluate the long‐term stability theoretically. On the other hand, considering practical applications, Wang et al. reported that the output amplitude of DC‐TENG maintains 48% after 90 000 cycles,^[^ ^35^ ^]^ which is still less than the range of TENG output amplitude in this work. When the same triboelectric materials are applied, it is suggested that the measurement results of the proposed sensor will be robust after 90 000 cycles.

### 2.4 Designable Threshold Displacement

**Figure 5:**
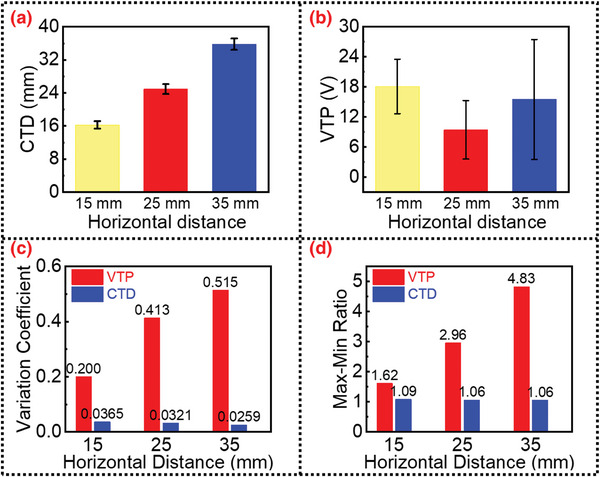
Sensing performances of the DC‐TENG sensors with different threshold displacements. a) CTD with different horizontal distance parameters; b) VTP with different horizontal distance parameters; c) comparison of variation coefficients between VTP and CTD; d) comparison of max–min ratios between VTP and CTD.

To satisfy the sensing demands in different practical scenarios, different displacement thresholds are needed. Therefore, robust and excellent performance at different specified thresholds is important for DC‐TENG displacement sensors. Previous theoretical analysis has shown that the threshold displacement of the DC‐TENG displacement sensor is approximately equal to the horizontal distance *L* between the CCE and FE. Therefore, sensors with different threshold displacements can be easily designed. In **Figure** **5**, the robustness test of different threshold displacements is conducted for sensors with horizontal distances *L* of 15, 25, and 35 mm, respectively.

As shown in Figure 5a, the CTDs of the three sensors are all stable under variable air humidity and roughness. The air humidity and roughness control experiments are set the same as those described in Section 2.3. Their average threshold displacements are 16.19, 24.94, and 35.81 mm when the horizontal distances of the TENG are 15, 25, and 35 mm, respectively. The threshold displacement and horizontal distance parameters are very close, indicating the convenience of sensor design. In contrast, Figure 5b shows that the VTPs of the three sensors all have large fluctuations in the aforementioned robustness test. In Figure 5c,d, the statistical analysis of robustness performances is presented. For the three DC‐TENG sensors with different threshold displacements, the variation coefficients of the CTD are only 2.45%, 3.21%, and 2.59% when the horizontal distances of the TENG are 15, 25, and 35 mm, respectively. The max–min ratios are 1.09, 1.06, and 1.06, respectively, which are close to 1, indicating a robust performance. On the contrary, for the VTPs, the variation coefficients and the max–min ratios are always unsatisfactory. These results demonstrate that the proposed robust displacement sensing method not only aids the design of sensors with different threshold displacements but also exhibits better robustness than the traditional amplitude‐based sensors. More information about the statistics data of Figure 5 is presented in Table S1, Supporting Information.

### 2.5 Robust Maximum Displacement Sensing

**Figure 6:**
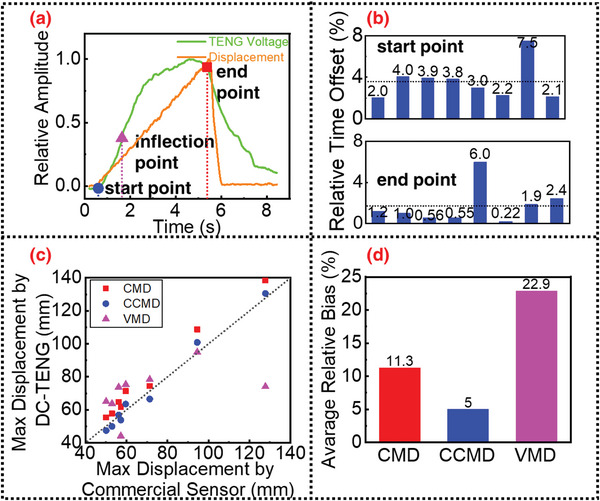
Sensing performances for the maximum displacement. a) Starting, inflection, and ending points obtained by the algorithm; b) sensing errors of the algorithm for the starting and ending points; c) character‐based, compensated character‐based, and voltage‐based maximum displacement (CMD/CCMD/VMD) compared to the standard commercial sensor; d) is a statistical analysis of (c).

As mentioned in the previous mechanism analysis, the proposed DC‐TENG sensor and intelligent algorithm can not only determine whether the displacement exceeds the threshold value or not but also sense the maximum displacement via the identification of the starting, inflection, and ending points. As shown in **Figure** **6**a, the feature points identified by the algorithm are very close to the real ones, which can be verified by the calibration results of standard commercial sensors. The recognition accuracy of the threshold displacement points is presented in Figure 4. Similarly, Figure 6b shows the sensing errors of the algorithms for the starting and ending points. The average errors of the starting and ending points are 3.6% and 1.7%, respectively, and the maximum errors are 7.5% and 6%, respectively. Further, the robustness of the proposed character‐based maximum displacement (CMD) can be verified by comparing it with the voltage‐based maximum displacement (VMD). For VMDs in Figure 6c,d, the signal amplitude is converted to displacement with a set of ideally best coefficients, which is unpredictable for practical application scenarios. Therefore, the comparison shown in Figure 6c,d is not fair, but even more biased toward VMD. However, the sensing accuracy of the CMD still easily outperforms VMD. The average relative bias is defined as,

(5) $$B=\frac{1}{N}\sum_{n=1}^{N}\frac{D\left(n\right)-D_{0}}{D_{0}}$$

where *D*_0_ is the true displacement. According to the statistical analysis of Figure 6d, compared to the VMD, the CMD reduces the average relative error from 22.9% to 11.3%.

In addition, if the sensing offset of the starting point in CMD is further compensated, marked as compensated character‐based maximum displacement (CCMD), such a sensing accuracy advantage can be further improved. Based on the experimental data, it is inferred that the sensing of the starting point, as shown in Figure 6b, is likely to have a positive bias compared with the true starting point; that is, it occurs later than the real starting point. By compensating for this positive bias, a more accurate maximum displacement sensing is obtained. As shown in Figure 6c,d, the average relative bias is further reduced to 5%. These results show that the proposed DC‐TENG displacement sensor has the advantage of robust performance not only in threshold displacement sensing but also in maximum displacement sensing.

## 3 Conclusion

Based on the DC‐TENG, a displacement sensor that is robust to environment‐related variable charge density is proposed. With the help of an intelligent signal processing algorithm, the accurate sensing of the threshold and maximum displacements can be realized according to the characteristics rather than the amplitude of the DC‐TENG signal. The threshold displacement being used as a reference point in principle is determined only by the structural parameters of the DC‐TENG. Therefore, the sensing robustness is achieved, avoiding influences from various environmental factors. The experimental results show that when the air humidity changes from 10% to 50% or the surface treatment power of the friction layer changes from 10% to 90%, compared with the traditional amplitude‐based displacement sensing, the proposed character‐based displacement sensing has significant advantages in variation coefficient and other consistency indicators. In addition, it is easy to design sensors with specific threshold displacement specifications for different application scenarios by changing the structural parameters. Overall, the results of this study provide a new technical route for designing robust self‐powered displacement sensors that can be applied to variable harsh environments.

## 4 Experimental Section

### Device Fabrication and Real‐Time Displacement Calibration

This robust displacement sensor device adopted the same air‐breakdown DC‐TENG structure as that used in previous literature.^[^ ^25^ ^]^ The negative TL was fluorinated ethylene propylene (FEP), the CCE and the FE were both Cu, and the surface of the FE used nitrile butadiene rubber (NBR) as the positive tribo‐layer. The threshold displacement of the sensor was modulated by the horizontal distance between the CCE and the FE. The strength of air breakdown was modulated by precisely controlling the distance between the CCE and the FEP via the thread. A commercial displacement sensor (MPS‐S‐1000MM‐V1, MIRAN Corporation) was used as the reference for calibrating the real‐time displacement of the DC‐TENG sensor. A linear motor (FBL80E500, FUYU Corporation) was used to generate the displacement. An electrometer (6514, KEITHLEY Corporation) was used to simultaneously measure the voltage signals of the DC‐TENG sensor and the commercial sensor.

### Air Humidity and Surface Roughness

The humidity robustness experiments were carried out via the test system as shown in Figure S3, Supporting Information. The air humidity was controlled by a dehumidifier (DH‐504B, CHKAWAI Corporation) and a humidifier (SC‐3G40A/B, MIDEA Corporation). The humidity value was measured in real‐time using a hygrometer (JR913, ANYMETRE Corporation). The experiments covered a humidity range from 10% to 50%, and the measured humidity data with time is presented in Figure S4, Supporting Information.

The roughness of the FEP tribo‐layer surface was controlled by plasma treatment (PTL‐VM500, PTL Corporation) with different powers. The duration of the plasma treatment was consistent with the standard procedure of the equipment. The power of plasma treatment in the experiment covered ≈0–90% of its rated power, which was 300 W. Surface roughness and 3D topography were measured by atomic force microscopy (Diension Icon, Bruker Corporation). The relative roughness and 3D topography data are presented in Figures S5 and S6, Supporting Information.

### Statistical Analysis

1. Pre‐processing of data: basal filtering was applied, including mean value based on sliding windows and sampling at specific intervals.
2. Data presentation: in Figure 5a,b, CTD and VTP are presented with mean ± 1.5 standard deviation; in Figure 6d, average relative bias is presented with the mean value.
3. Sample size (*n*) for each statistical analysis: in Figure 3 a Figure S2, Supporting Information, the voltage waveform is presented with the average window of 50 points (*d* = 5 mm) and 25 points (*d* = 10, 20, 30 mm); in other calculation and analysis, the voltage waveform is processed by an average of 100 points and sampling intervals of 20 points.
4. Statistical methods used to assess significant differences with sufficient details: uninvolved.
5. Software used for statistical analysis: MATLAB 2016b and OriginLab 2018.

## Conflict of Interest

The authors declare no conflict of interest.

## Supporting information

Supporting information

## Data Availability

The data that support the findings of this study are available from the corresponding author upon reasonable request.
