# Supplementary material for: Robust Displacement Sensing by Direct‐Current Triboelectric Nanogenerator Via Intelligent Waveform Recognition
Source: Adv Sci (Weinh). 2022 Dec 4;10(4):2204694. doi: 10.1002/advs.202204694 (PMC9896052; doi:10.1002/advs.202204694)
Supplement: Supplementary file 1 — Supporting information [file ADVS-10-2204694-s001.pdf]

ADVANCED SCIENCE
Open Access

Supporting Information

for *Adv. Sci.*, DOI 10.1002/advs.202204694

Robust Displacement Sensing by Direct-Current Triboelectric Nanogenerator Via Intelligent Waveform Recognition

Keren Dai, Xuyi Miao, Wenling Zhang, Xiaohua Huang, He Zhang and Sang-Woo Kim**

Supporting Information

Robust Displacement Sensing by Direct-Current Triboelectric Nanogenerator via Intelligent Waveform Recognition

Keren Dai[#], Xuyi Miao[#], Wenling Zhang, Xiaohua Huang [*], He Zhang, Sang-Woo Kim[*]

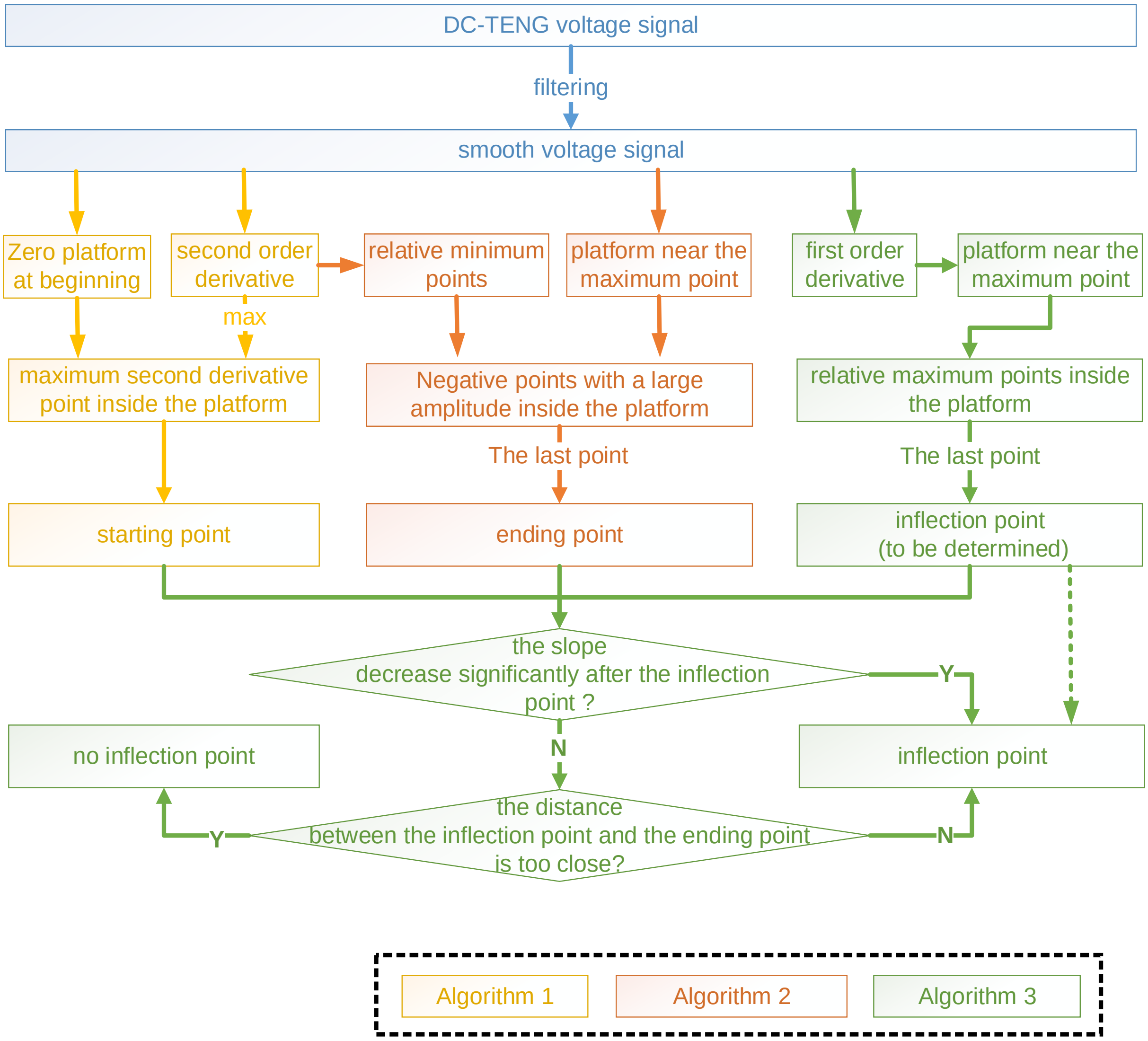

Figure S1. Detailed signal processing algorithm for recognition of feature points. Algorithms 1, 2, and 3 for the starting, ending, and inflection points, respectively.

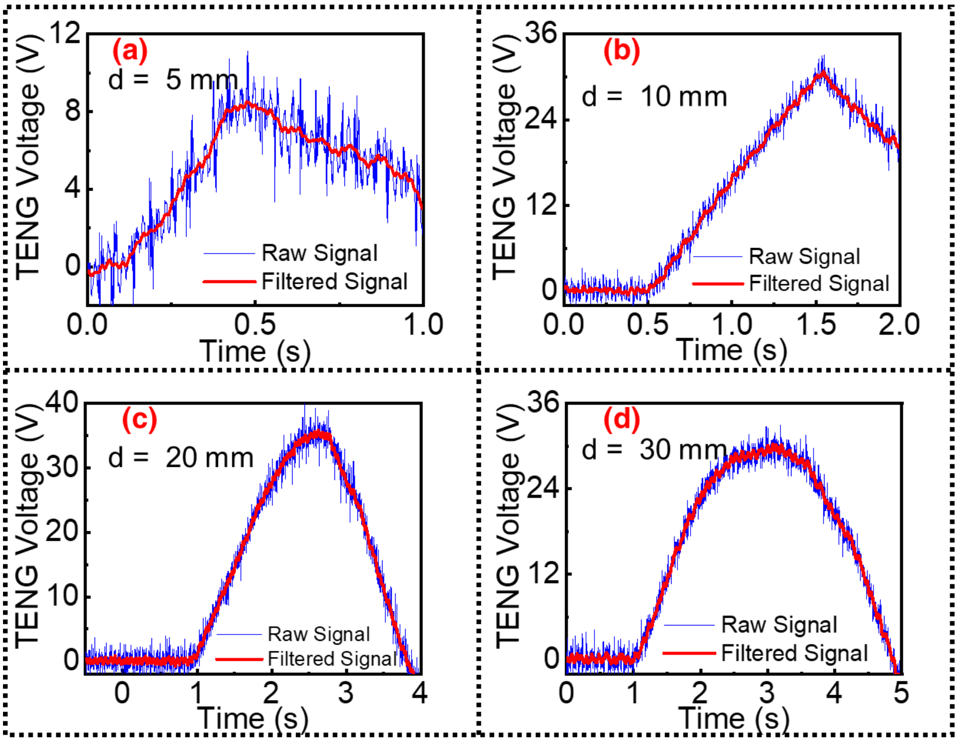

Figure S2. Raw voltage signals and filtered voltage signals of the DC-TENG sensors with different maximum displacements. (a) Maximum displacement is 5 mm, less than the threshold; (b) Maximum displacement is 10 mm, less than the threshold; (c) Maximum displacement is 20 mm, larger than the threshold; (d) Maximum displacement is 30 mm, larger than the threshold.

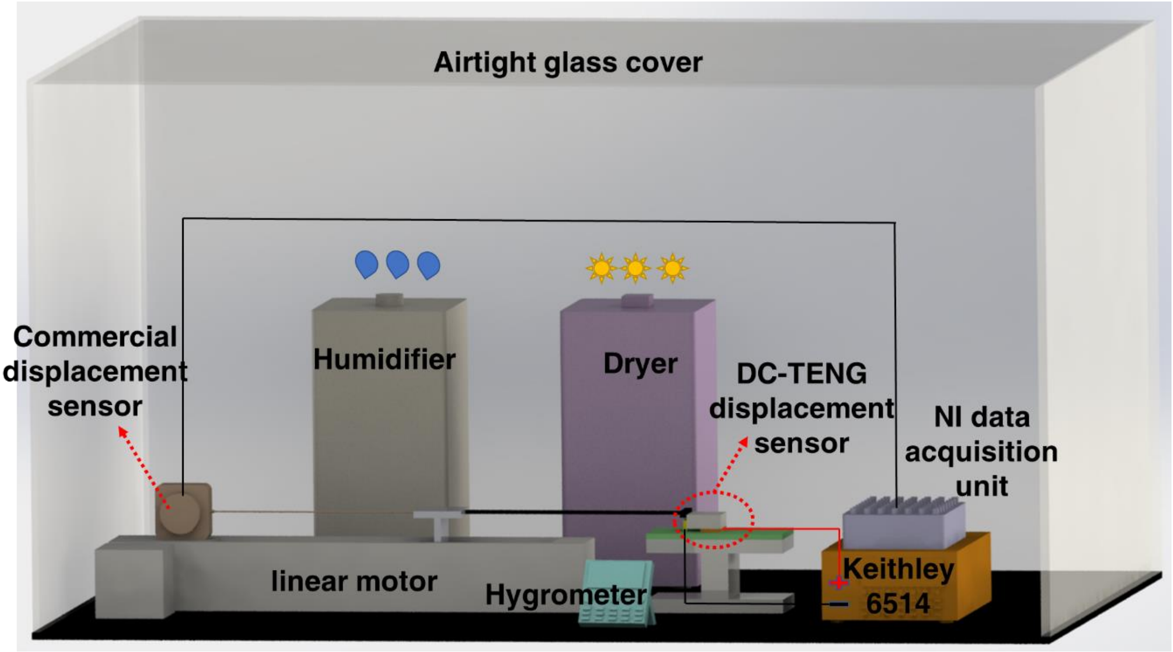

Figure S3. Experimental system for humidity robustness.

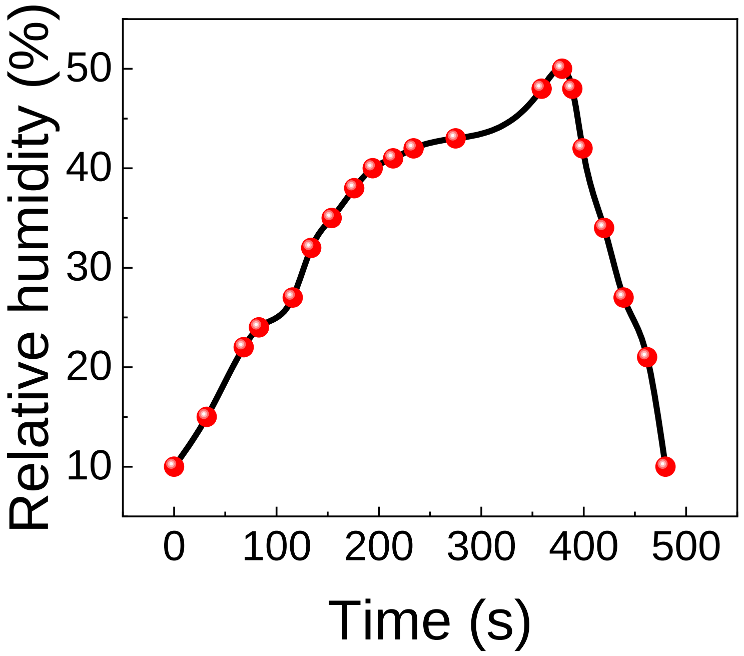

Figure S4. Measured humidity with time (while it changes from 10% to 50% and then back to 10%).

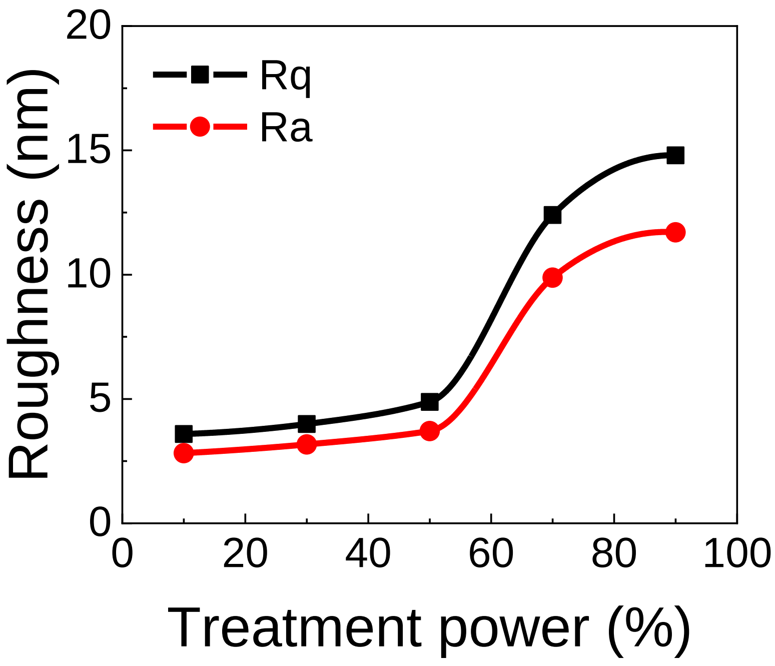

Figure S5. Measured roughness of the friction layer with different plasma treatment power. Rq and Ra refer to the arithmetical mean and root-mean-square values, respectively.

(a) 10% Treatment power

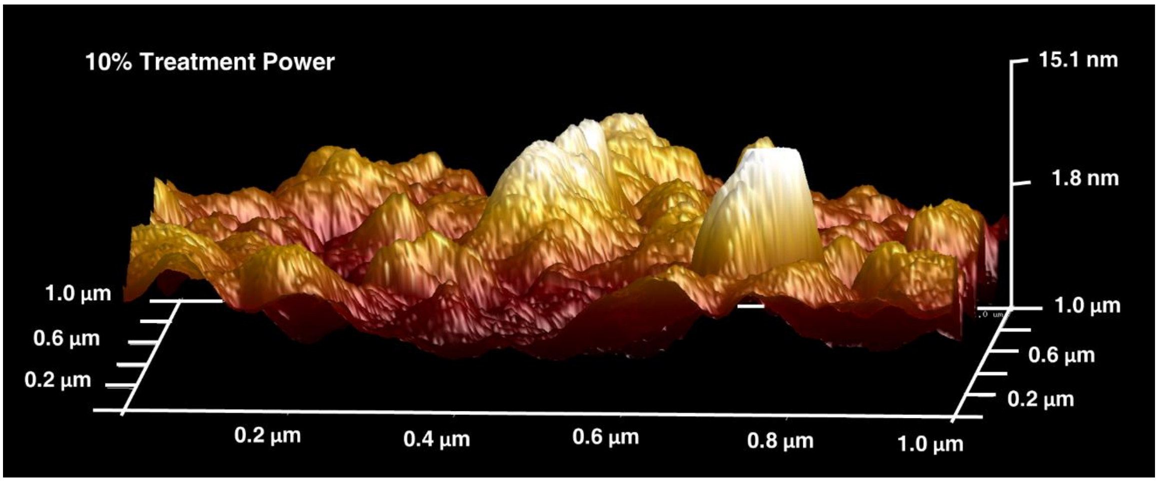

(b) 30% Treatment power

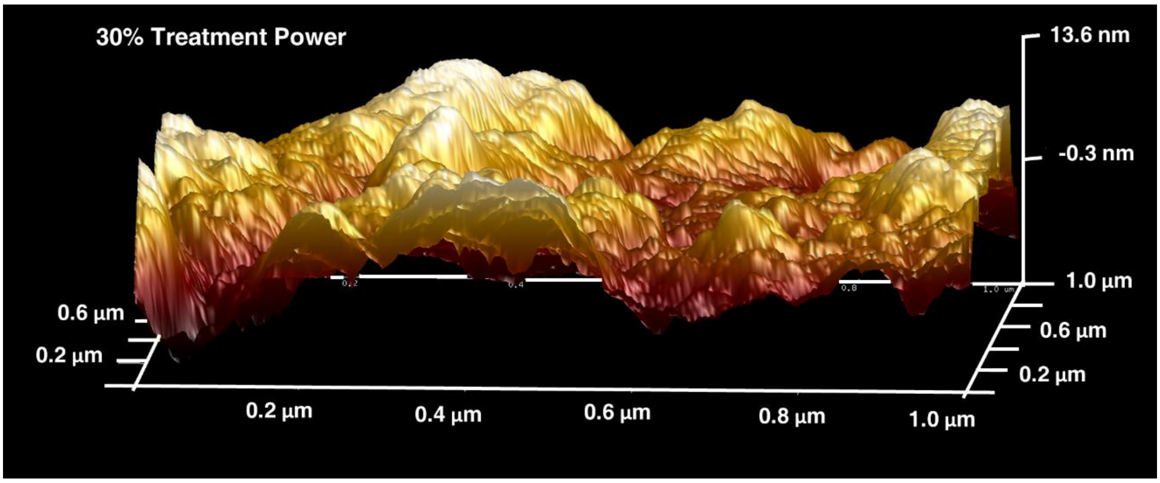

(c) 50% Treatment power

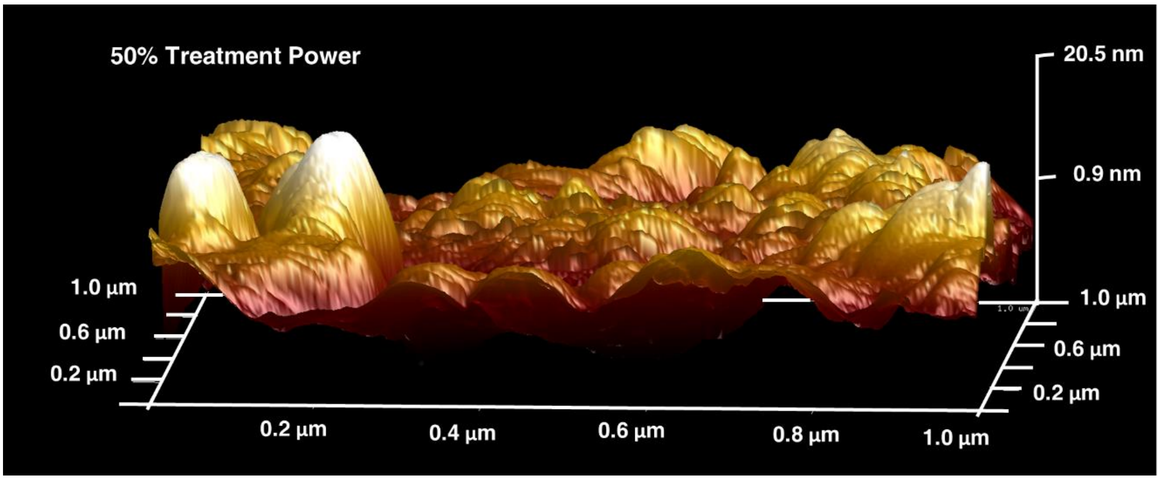

(d) 70% Treatment power

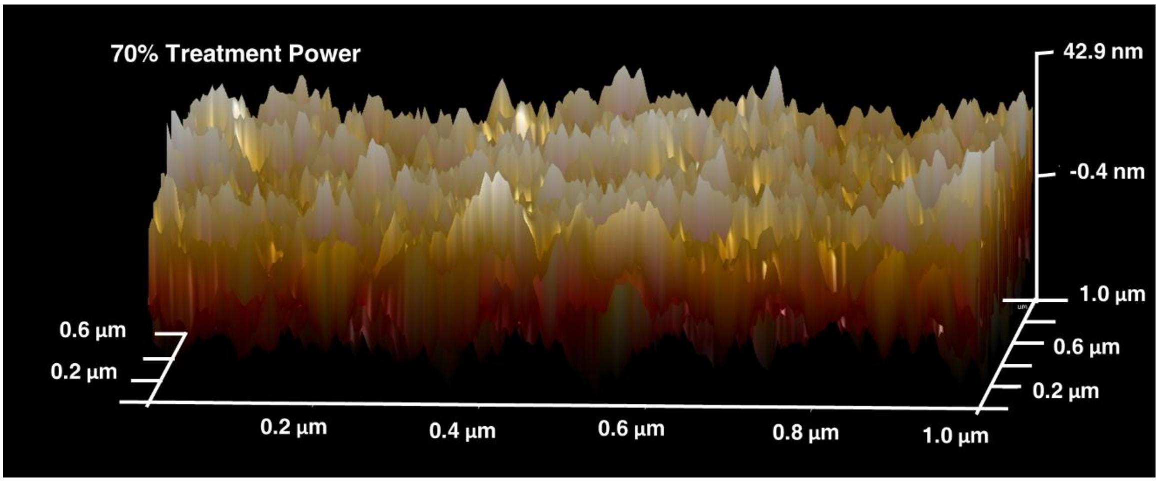

(e) 90% Treatment power

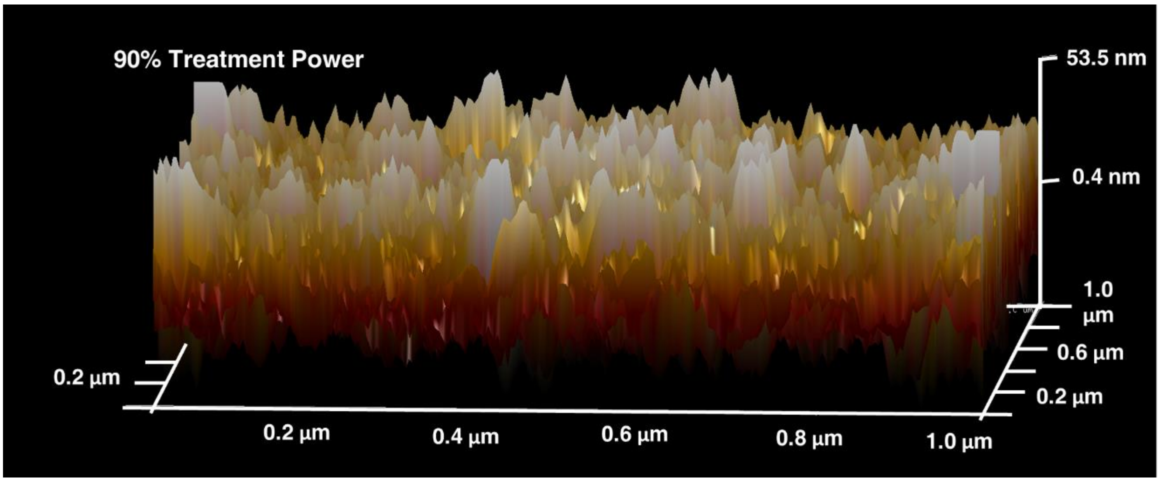

Figure S6. Three-dimensional surface topography of the friction layer with different plasma treatment power.

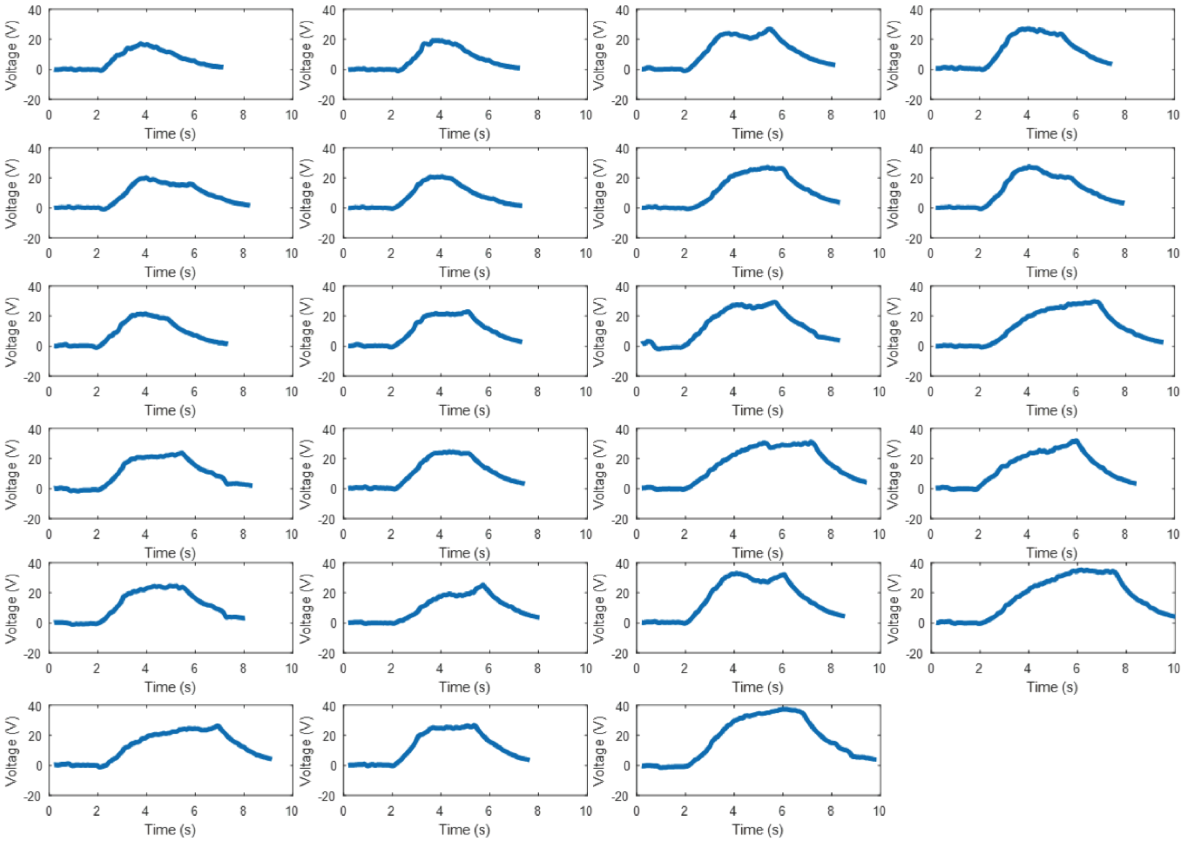

Figure S7. Raw data in Figure 4 with humidity ranged from high to low.

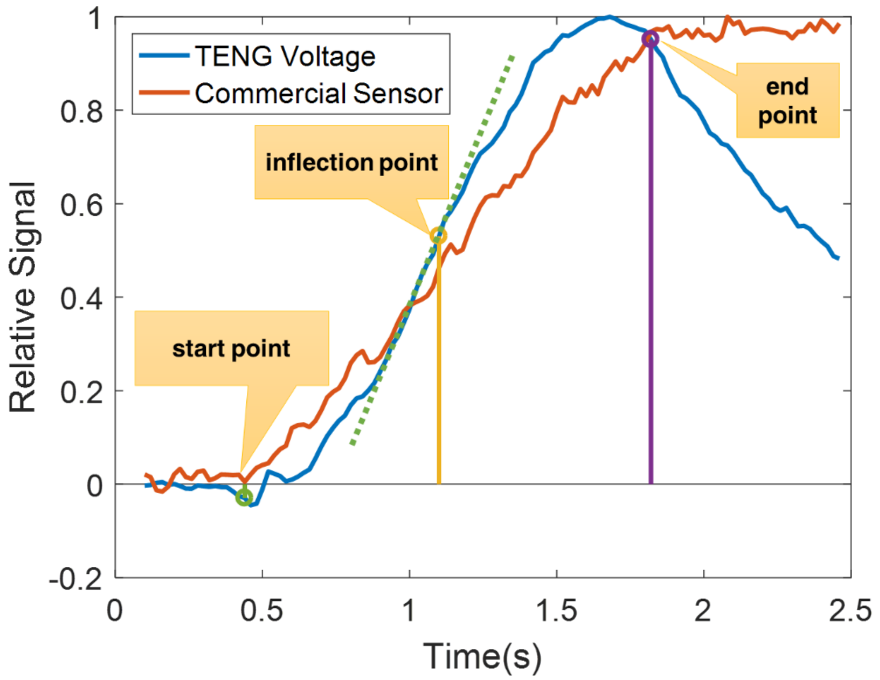

Figure S8. The Output displacement sensing signal of DC-TENG at 60% humidity and its corresponding feature point recongnition.

Table S1. Detailed statistics date of VTP and CTD with different threshold displacements via specific horizontal distance design at 15, 25, and 35 mm.

Issue		Average	Standard Deviation	Variation Coefficient	Min Value	Max Value	Max-Min Ratio
VTP	L = 15 mm	15.09	3.02	0.20	11.74	18.97	1.62
	L = 25 mm	7.85	3.25	0.41	4.03	11.95	2.96
	L = 35 mm	12.93	6.65	0.51	3.88	18.77	4.83
CTD	L = 15 mm	8.13	0.30	0.04	7.85	8.55	1.09
	L = 25 mm	12.47	0.40	0.03	12.05	12.82	1.06
	L = 35 mm	17.90	0.46	0.03	17.25	18.31	1.06
